# Supplementary material for: Epigenomic landscape of single vascular cells reflects developmental origin and disease risk loci
Source: Mol Syst Biol. 2025 Sep 10;21(11):1522–46. doi: 10.1038/s44320-025-00140-2 (PMC12583710; doi:10.1038/s44320-025-00140-2)
Supplement: Supplementary file 1 — Appendix [file 44320_2025_140_MOESM1_ESM.pdf]

Appendix for *Epigenomic landscape of single vascular cells reflects developmental origin and disease risk loci*

Chad S. Weldy, Soumya Kundu, João Monteiro, Wenduo Gu, Albert J. Pedroza, Alex R. Dalal, Matthew D. Worssam, Daniel Li, Brian Palmisano, Quanyi Zhao, Disha Sharma, Trieu Nguyen, Ramendra Kundu, Michael P. Fischbein, Jesse Engreitz, Anshul B. Kundaje, Paul P. Cheng, Thomas Quertermous

Table of Contents:
Appendix Figure S1: page 2
Appendix Figure S2: page 3
Appendix Figure S3: page 4
Appendix Figure S4: page 5
Appendix Figure S5: page 6
Appendix Figure S6: page 7
Appendix Figure S7: page 8

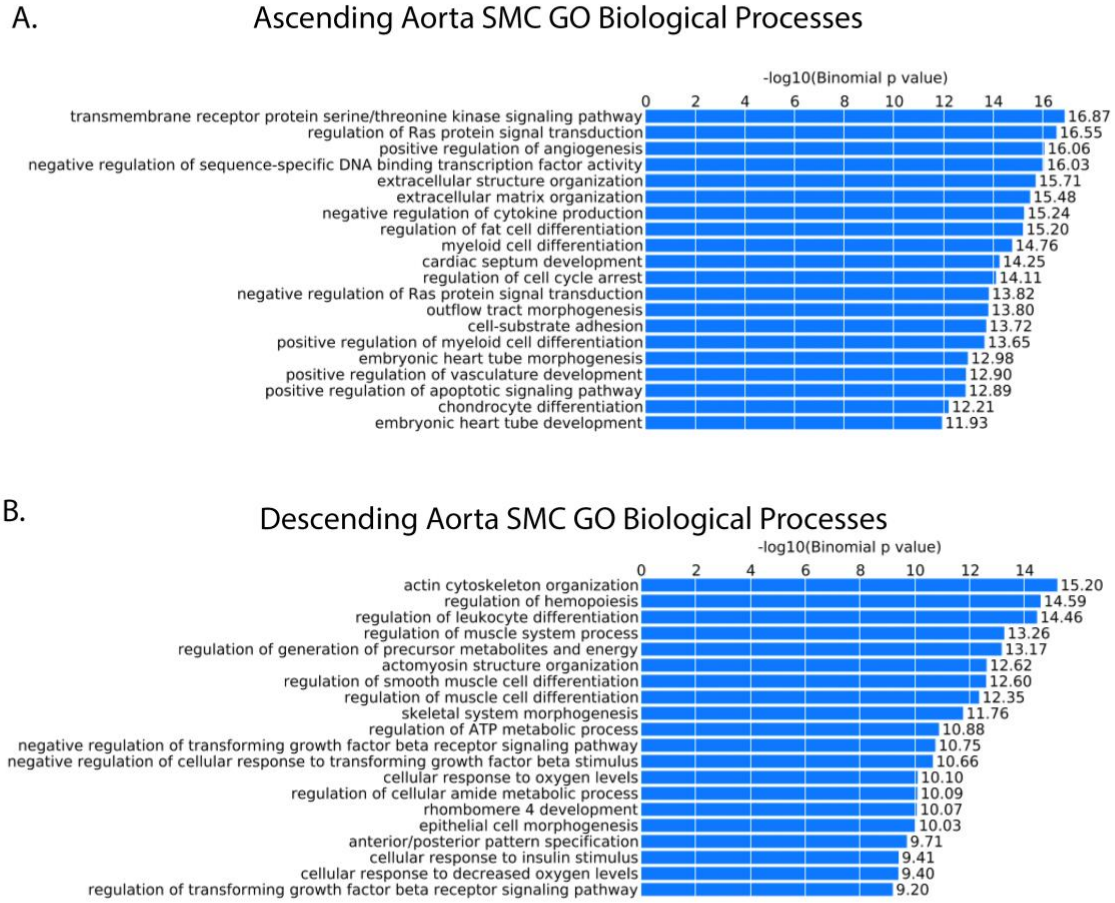

Appendix Figure S1. Top biological processes from GREAT for peaks marking SMCs isolated from the ascending aorta (A) and descending aorta (B).

A.

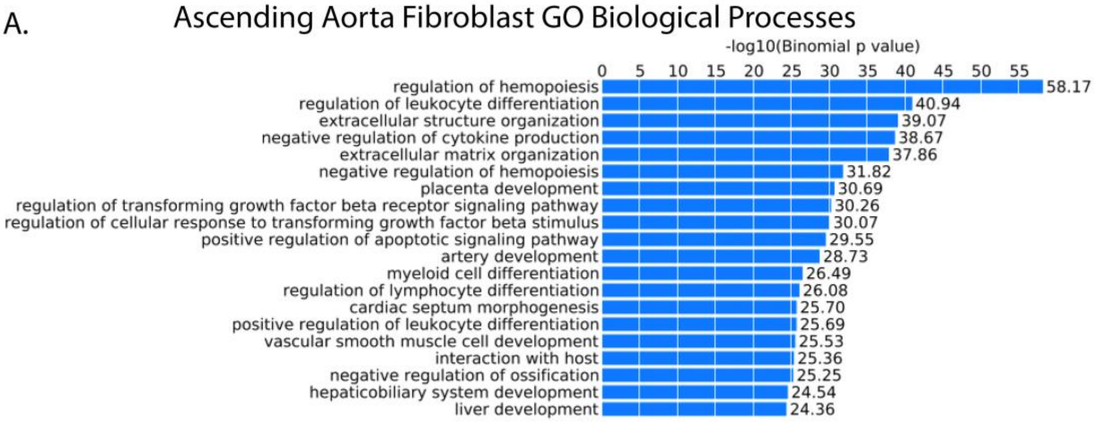

B.

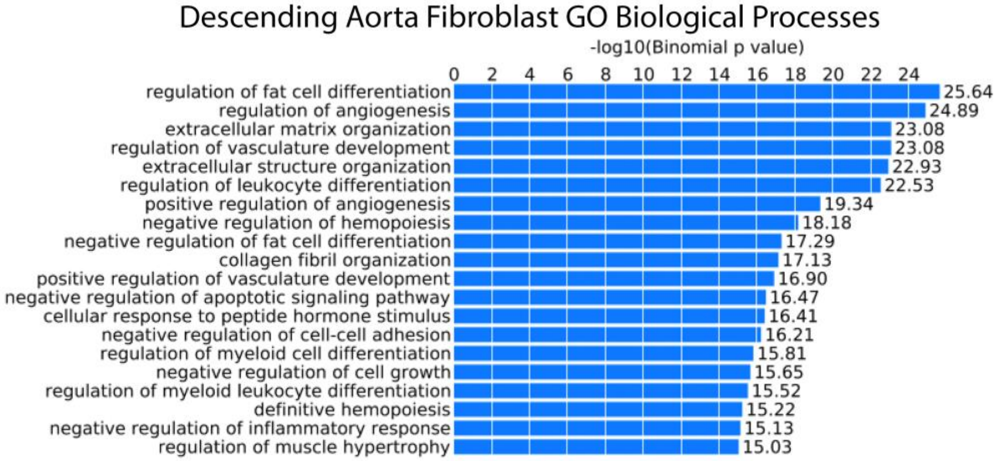

Appendix Figure S2. Top biological processes from GREAT for peaks marking fibroblasts isolated from the ascending aorta (A) and descending aorta (B).

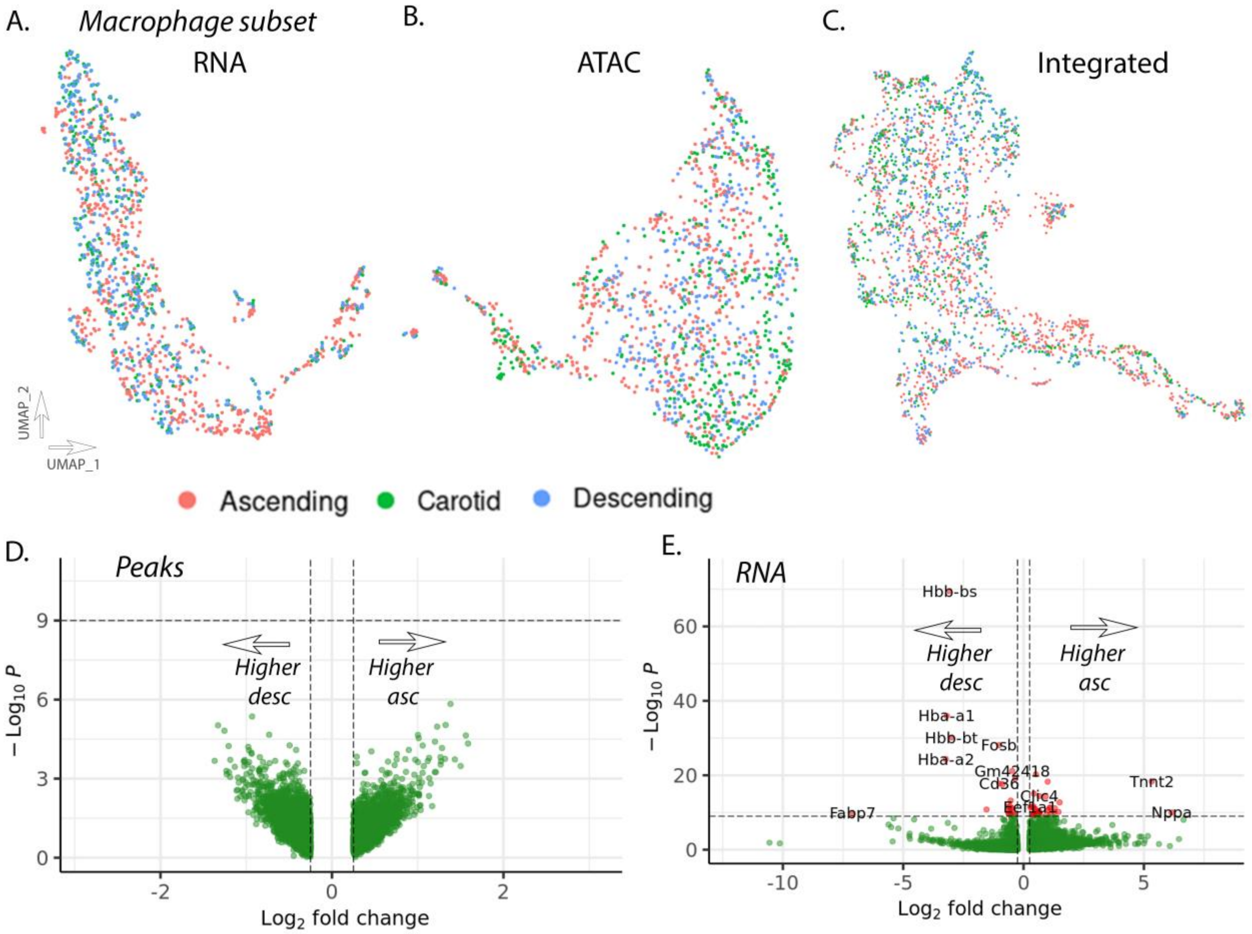

Appendix Figure S3. Single cell transcriptomic and epigenomic analysis of macrophage cells reveals notable homogeneity between vascular sites. UMAP visualization of macrophage cell subset for RNA (A), ATAC (B), and integrated (C) datasets. Volcano plots for differential peak accessibility (D) and RNA expression (E).

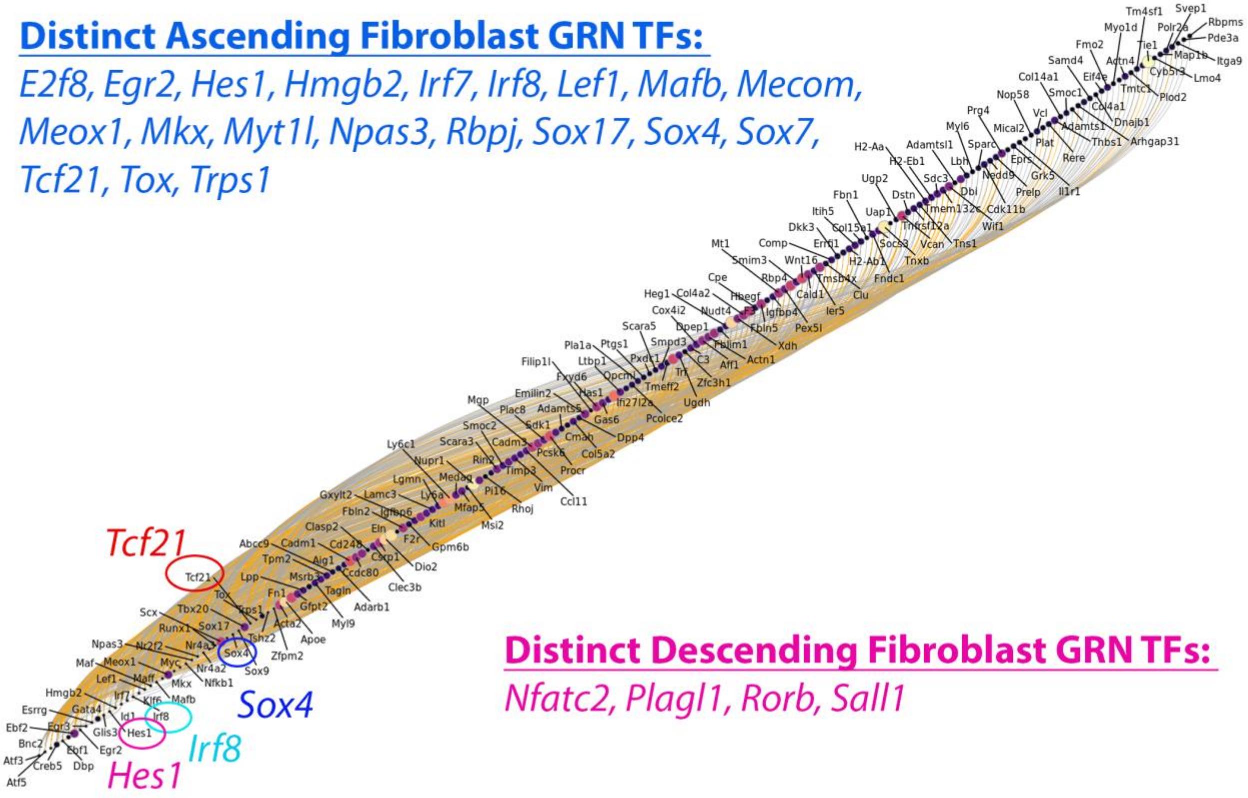

Appendix Figure S4. Distinct ascending fibroblast gene regulatory networks. GRN visualization for ascending fibroblasts network analysis, where each dot represents gene and/or TF, color represents regulatory strength (darker with higher strength), and size of dot reflects degree of centrality.

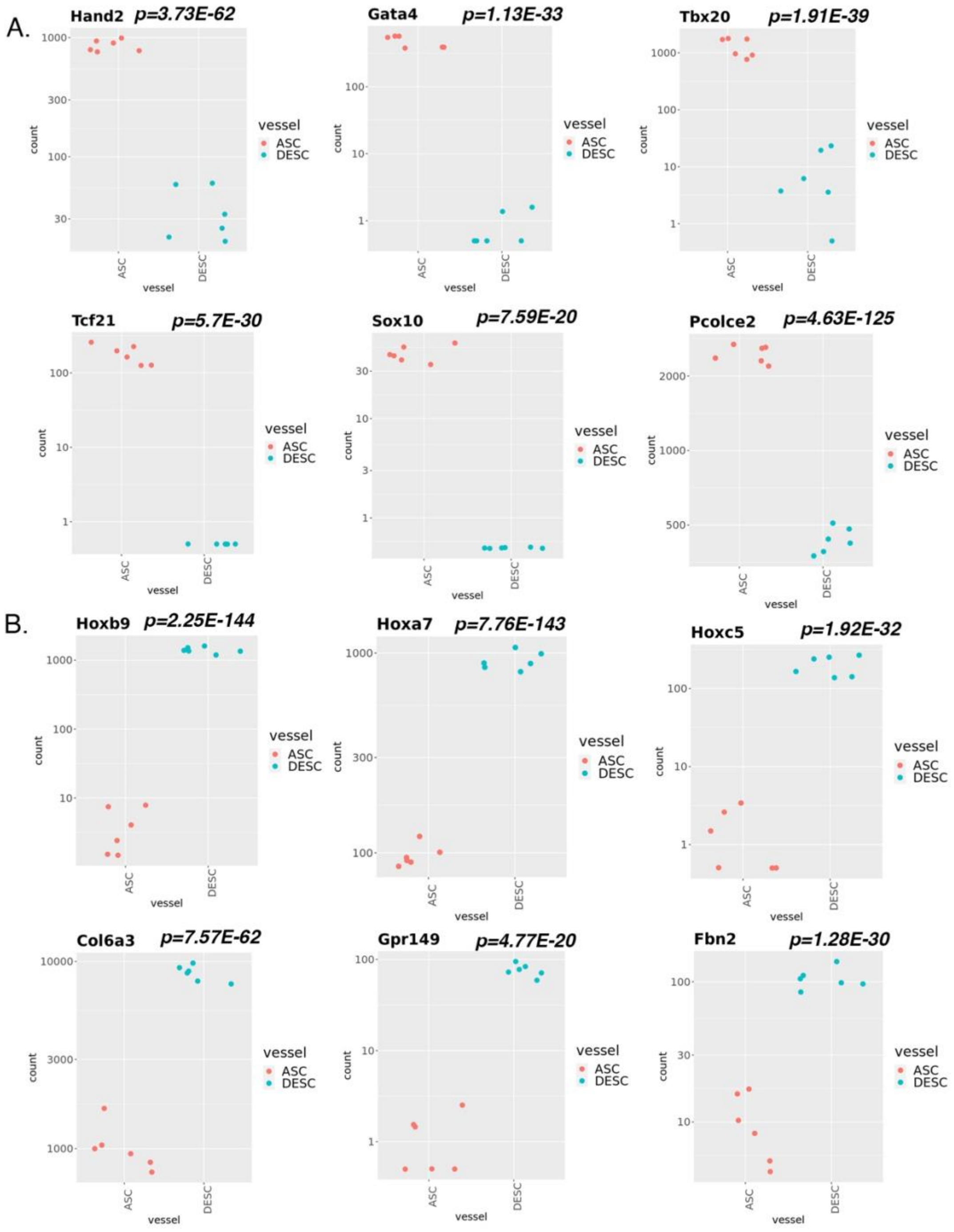

Appendix Figure S5. RNA expression from primary adventitial fibroblast in vitro culture highlighting genes with increased expression in ascending fibroblasts including *Hand2*, *Gata4*, *Tbx20*, *Tcf21*, *Sox10*, and *Pcolce2* (A), and increased expression in descending fibroblasts including *Hoxb9*, *Hoxa7*, *Hoxc5*, *Col6a3*, *Gpr149*, and *Fbn2* (B). Y-axis represents normalized read counts by DESeq2 shown on log10 scale. P-value for comparison shown for each gene.

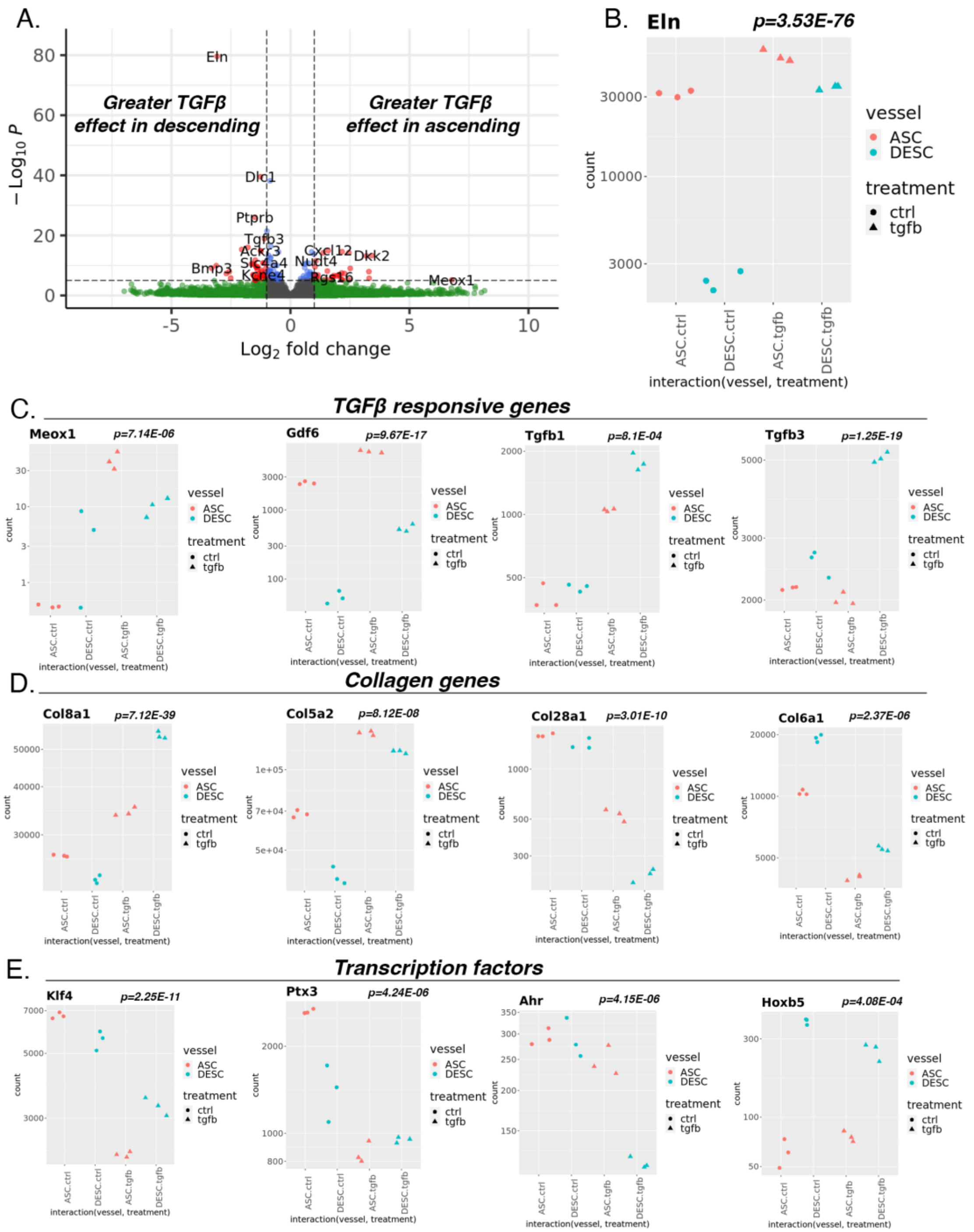

Appendix Figure S6. Volcano plot of interaction analysis of RNAseq data revealing genes with differential response to TGFβ by vascular site (log2FC > 0 represents greater effect TGFβ in ascending; log2FC < 0 represents greater effect TGFβ in descending) (A). RNAseq expression for top interaction gene *Eln* (B). RNAseq expression for TGFβ responsive genes *Meox1*, *Gdf6*, *Tgfb1*, and *Tgfb3* (C) collagen genes *Col8a1*, *Col5a2*, *Col28a1*, *Col6a1* (D) and transcription factors *Klf4*, *Ptx3*, *Ahr*, and *Hoxb5* (E). Y-axis represents normalized read counts by DESeq2 shown on log10 scale. P-value for interaction shown for each gene.

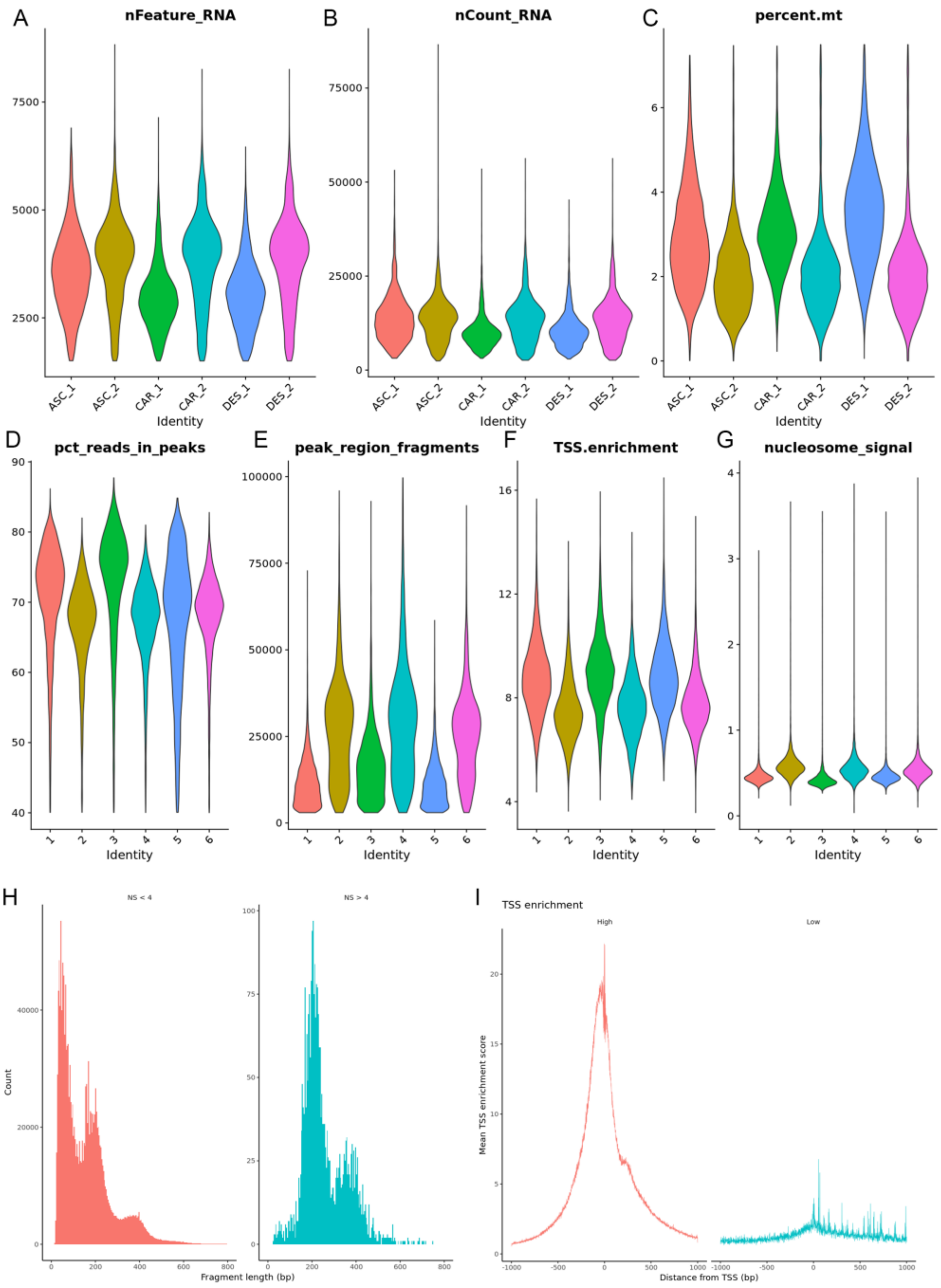

Appendix Figure S7. QC metrics for scRNAseq and scATACseq datasets. For scRNAseq datasets, violin plots for nFeature_RNA (A), nCount_RNA (B), and percent.mt (C) across 6 captures. For scATACseq datasets, violin plots for pct_reads_in_peaks (D), peak_region_fragments (E), TSS.enrichment (F), and nucleosome_signal (G). Fragment histogram of region chr1-1-10000000 in combined aggr dataset based on low and high nucleosome signal (NS <4, left, NS >4, right) (H). TSS plot for combined aggr dataset based on high and low TSS enrichment (TSS >2, left, TSS <2, right).
